# Application of POCUS in patients with COVID-19 for acute respiratory distress syndrome management: a narrative review

**DOI:** 10.1186/s12890-022-01841-2

**Published:** 2022-02-05

**Authors:** Xuehui Gao, Xiaojing Zou, Ruiting Li, Huaqing Shu, Yuan Yu, Xiaobo Yang, You Shang

**Affiliations:** 1 Department of Critical Care Medicine, Union Hospital, Tongji Medical College, Huazhong University of Science and Technology, No.1277, Jiefang Avenue, Wuhan, 430022 Hubei People’s Republic of China; 2 Institute of Anesthesiology and Critical Care Medicine, Union Hospital, Tongji Medical College, Huazhong University of Science and Technology, Wuhan, 430022 Hubei People’s Republic of China

**Keywords:** COVID-19, SARS-CoV-2, Acute respiratory distress syndrome, ARDS, Point-of-Care ultrasound, POCUS

## Abstract

COVID-19 has inflicted the world for over two years. The recent mutant virus strains pose greater challenges to disease prevention and treatment. COVID-19 can cause acute respiratory distress syndrome (ARDS) and extrapulmonary injury. Dynamic monitoring of each patient's condition is necessary to timely tailor treatments, improve prognosis and reduce mortality. Point-of-care ultrasound (POCUS) is broadly used in patients with ARDS. POCUS is recommended to be performed regularly in COVID-19 patients for respiratory failure management. In this review, we summarized the ultrasound characteristics of COVID-19 patients, mainly focusing on lung ultrasound and echocardiography. Furthermore, we also provided the experience of using POCUS to manage COVID-19-related ARDS.

## Background

The coronavirus disease 2019 (COVID-19), caused by severe acute respiratory syndrome coronavirus 2 (SARS-CoV-2), is highly transmittable and potentially lethal [1, 2]. Although most patients with COVID-19 have mild manifestations, approximately 15% to 30% of hospitalized patients deteriorate, requiring admission to an intensive care unit (ICU) or invasive mechanical ventilation for acute respiratory distress syndrome (ARDS) [3], which seriously affects their prognosis [4, 5]. However, some experts suggested that the COVID-19-related ARDS was somewhat different from classical ARDS, because of a relatively preserved compliance of the respiratory system despite marked hypoxemia [6–8]. Furthermore, the highly contagious nature of SARS-CoV-2 also increases management difficulties. Point-of-care ultrasound (POCUS)—a convenient bedside tool for monitoring pulmonary function and assessing related complications—is considered as a valid aid in COVID-19 patient management [9]. At present, there are few summaries on POCUS guiding the management of COVID-19-related ARDS. In this review, we will summarize the ultrasonographic characteristics of COVID-19 patients, mainly focusing on the lung ultrasound and echocardiography. Furthermore, we also provided the experience of using POCUS to manage COVID-19-related ARDS (Table 1).

**Table 1:** Summarizing the different uses of the different POCUS modalities in COVID-19 related ARDS

POCUS modalities	The different uses of the different POCUS modalities
Lung ultrasound	1. Allowing to rule in or rule out COVID‑19 pneumonia combined with a medical history during the pandemic [17]
	2. Screening the patient and aiding precise triage and treatment allocation in both the emergency and ICU departments
	3. Assessing the lung lesions' progress and evaluating the severity of COVID-19 [10, 12, 18, 19]
	4. Combing with echocardiography, diaphragmatic ultrasound and other aspects to guide the comprehensive treatments for COVID-19 related ARDS and monitor the response to treatments
Echocardiography	1. Identifying serious RV complications such as ACP, pulmonary embolism, pulmonary hypertension, etc. [29–33, 73]
	2. Detecting other complications related to LV dysfunction [30]
	3. Guiding the comprehensive treatments for COVID-19 related ARDS, mainly the management of cardiac complications and hemodynamics
Other aspects	1. IVC ultrasound combined with LUS and echocardiography is used for fluid resuscitation administration [79]
	2. Thoracic and abdominal ultrasounds help detect free fluid, empyema, pneumothorax, or cardiac tamponade
	3. Vascular ultrasound helps identify DVT and guide catheterization
	4. Obstetric ultrasound helps manage pregnant women infected with COVID-19 [39]

## Main text

### POCUS in COVID-19-related ARDS

Ultrasonography, a safe and effective form of imaging, has been used by physicians to aid in diagnosis and guide procedures for more than half a century. Over the past two decades, ultrasound equipment has become more compact, higher functional, and less expensive, which has facilitated the growth of POCUS, an ultrasound performed and interpreted by the clinician at the bedside. It is this feature that lead to the broad applications of POCUS in the ongoing battle against the COVID-19 pandemic [10–12].

### Lung ultrasound

The SARS-CoV-2 infection mainly affects the respiratory system. Although chest CTs are the gold standard for lung imaging, a systematic CT scan during the pandemic is not feasible for many reasons, such as the risk of SARS-CoV-2 transmission when infected patients are transferred to radiology facilities. Thus, Lung ultrasound (LUS) was validated for lung aeration monitoring in ARDS patients with a good correlation with chest CTs [13]. A retrospective comparison study demonstrated that LUS is more sensitive than chest CT to detect lesions in COVID-19 patients [14].

LUS can rapidly define alterations affecting the ratio between tissue and air in the superficial lung by trained clinicians. COVID-19 pneumonia imaging typically shows bilateral multilobar ground-glass opacities or consolidative opacities with a peripheral, or posterior, distribution. These characteristics provide an ideal fundamentals for LUS [15, 16]. Recent multi-center studies showed that in patients suspected for COVID-19, LUS patterns of probability combined with a medical history allowed to rule in or rule out COVID-19 pneumonia at bedside with high accuracy [17]. In the early and mild infection stage, focal B lines are the main pattern; in the progressive stage and critically ill patients, thickened pleural line, multiple, or fused B-lines and subpleural non-homogeneous consolidations are the main features; in an advanced stage, the main features are multiple continuous large-scale hypoechoic consolidation with hepatization sign, diffused B-lines, and extensive air bronchogram sign [10, 12, 18]. Furthermore, pleural line thickening with uneven B lines is a notable pattern, suggesting that the patient may be complicated by pulmonary fibrosis, an important factor influencing pulmonary function in COVID-19 patients [18]. Additionally, recent studies proposed a more precise evaluation standard, namely LUS score (LUSS), and demonstrated that LUSS was correlated to disease severity and progression [19], and had a good agreement with COVID-19 patients outcomes [20–22].

LUS also has some limitations in COVID-19 and there are occasions in which chest CTs are needed. First, it should be highlighted that all lesions must be extended to the pleural surface to be detected by LUS, because examination of the central structures is prevented by the barrier created by the pleural-lung interface [23]. And consolidation has to be within an intercostal window. This means that a small number of cases will not be identified by LUS. Moreover, LUS is often unable to identify lesion chronicity, limiting its utility in a person with pre-existing pulmonary conditions [11]. Additionally, although the sensitivity of LUS is high, its specificity may not be the same. Especially in the post-epidemic era, it is difficult to distinguish CVOID-19 from other causes of pneumonia (atypical bacteria, flu viruses, etc.) [24].

### Echocardiography

About 20% of hospitalized patients and up to 67% of critically ill patients with COVID-19 will develop ARDS [5, 25]. Cardiac failure, in particular right ventricular (RV) dysfunction, is commonly encountered in moderate to severe ARDS. In the early stages of the pandemic, some reports have indicated that cardiac complications not only were common in COVID-19 infection but also are associated with increased mortality [26, 27]. The high mortality and yet unknown aspects of the COVID-19 clinical course necessitate cardiac evaluations.

The most frequent cardiac abnormality is RV dysfunction among patients with COVID-19 infection [28]. RV dysfunction by echocardiography is commonly defined as the presence of pressure and/or volume overload of the right ventricle [29]. RV volume overload is defined as RV dilation and RV pressure overload is defined as dyskinetic movement of the septum during end-systole. According to the American Society of Echocardiography RV dysfunction is present when the parameters to quantify RV function are less than the lower value of the normal range: tricuspid annular plane systolic excursion is less than 17 mm, pulsed Doppler S wave is less than 9.5 cm per second, RV fractional area change is less than 35%, RV ejection fraction is less than 45% [30]. In the RV-focused view, RV diameter greater than 41 mm at the base and greater than 35 mm at midlevel indicates chamber dilatation [30]. Clinically, a simpler and more intuitive evaluation method is to compare the right ventricular end diastolic area (RVEDA) with the left ventricular end diastolic area (LVEDA) [31, 32]. Right ventricular dilation is defined as moderate when RVEDA/LVEDA ratio is between 0.6 and 1, and as severe when this ratio is more than 1.0 [33]. Additionally, RV dilatation can cause shifting of the interventricular septum toward the left, the “D sign” also is a simple method to assess RV size.

Due to ‘ventricular interdependence’, RV dilatation can impede left ventricular diastolic filling, resulting in left ventricular (LV) dysfunction. Recently, A large study about Doppler echocardiography indicated that LV haemodynamics can aid in risk stratification of patients with severe COVID-19, and LV low output and elevated filling pressure are independent predictors of mortality [34]. Two-dimensional and three-dimensional echocardiography can realize real-time measurement of LV diameters, volumes, ejection fraction, and mass, and even finer measurement, including the peak early filling (E wave) and late diastolic filling (A wave) velocities, E/A ratio, lateral annular velocities (e′), and deceleration time of early filling velocity [30]. García-Cruz et al. proposed an algorithm, the ORACLE protocol, to perform a quick systemic evaluation of biventricular function, valvular heart disease, pericardial effusion, hemodynamics, and lung ultrasound using POCUS [35].

### Other aspects

Many critically ill COVID-19 patients develop secondary muti-organ dysfunction [36], including acute kidney injury (AKI), liver injury gastrointestinal disturbance, cardiac dysfunction, as well as hypercoagulability with thromboembolic events, such as ischemic stroke. These dysfunctions may be evaluated with POCUS. POCUS can easily identify post-renal and pre-renal causes of AKI by evaluating hemodynamics assessment and post-renal obstrucations. Also, it can assess acute gastrointestinal complications in COVID-19 patients, including cholestasis and bowel ischemia [37], and detect circulating microemboli within cerebral arteries using transcranial Doppler ultrasound [38]. In addition, ultrasonography has been used in obstetrics for decades, with no evidence of harmful effects at normal diagnostic levels. POCUS facilitate care for pregnant women with SARS-CoV-2 infection and their babies [39].

### Treatment for COVID-19-related ARDS Patients with POCUS

**Fig. 1:**
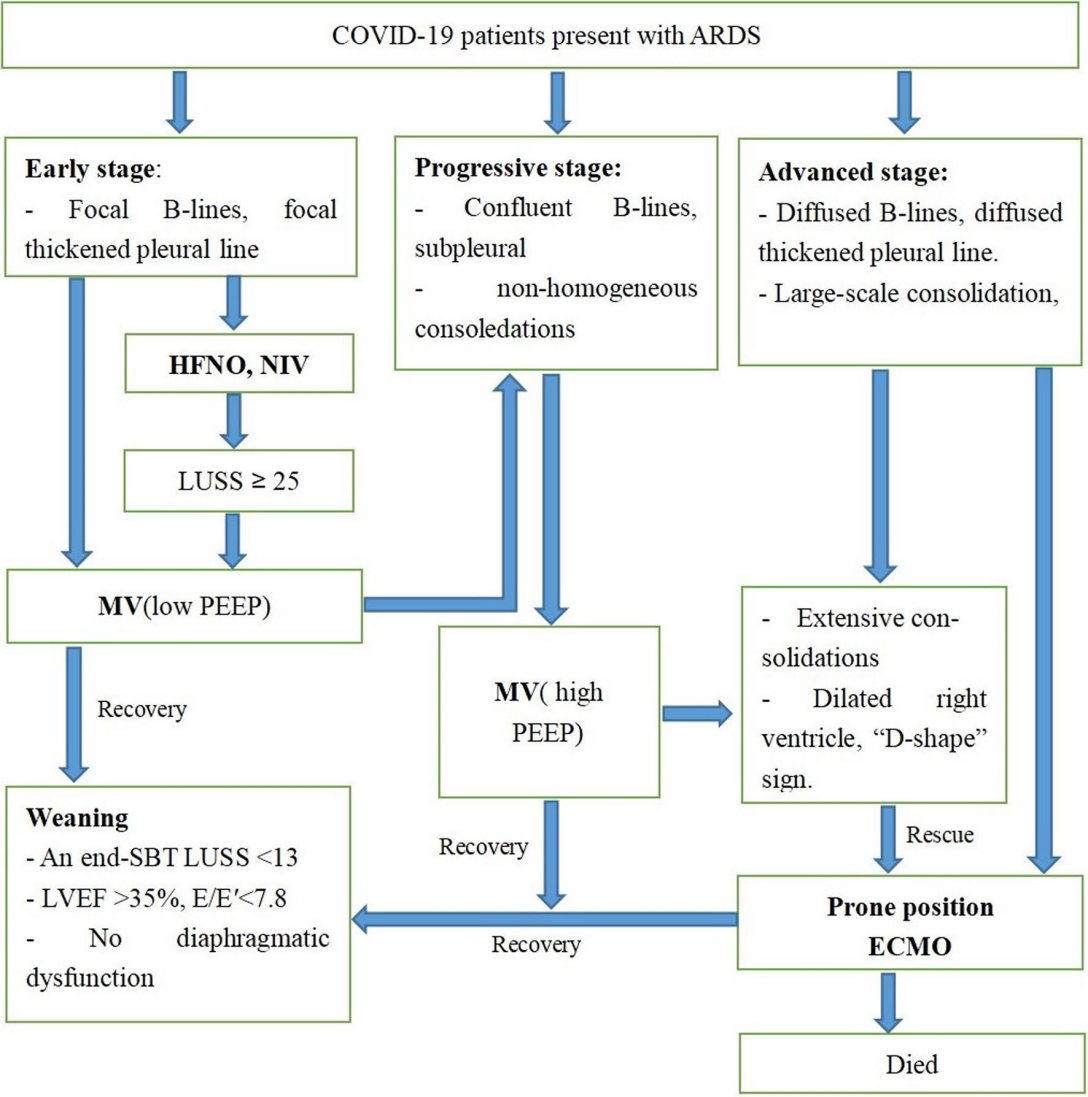
A road map for the possible comprehensive use of POCUS in COVID-19 ARDS. MV mechanical ventilation, PEEP positive end expiratory pressure, SBT spontaneous breathing trial, LUS lung ultrasound, LVEF left ventricular ejection fraction, E pulsed wave doppler early mitral valve inflow velocity, E′ mitral annular tissue doppler velocity, ECMO extracorporeal membrane oxygenation

POCUS is vital in COVID-19-related ARDS Patients because it provides real time information for diagnosis and treatment. Figure 1 presents a road map for the possible comprehensive use of POCUS in COVID-19 ARDS.

### Ventilation support and open-lung

The mainstream of treatment for COVID-19 patients is still supportive care. Ventilation support remains the most important aspect. However, ventilation strategies vary for patients at different stages of the disease. POCUS can be used to track the course of COVID-19 pneumonia and monitor the response to ventilation support.

High-flow nasal oxygen and non-invasive ventilation are reserved for patients in the early stage of ARDS [40]. However, considering rapid clinical deterioration in severe COVID-19 patients and self-inflicted lung injury caused by respiratory distress, delayed intubation may lead to emergent intubations and mortality. POCUS is a valuable tool for close and real-time monitoring of lung function, which can be used to determine the timing of invasive mechanical ventilation, reducing the exposure risk of health-care workers in emergencies. A recent study showed that a LUSS ≥ 25 on admission had 90% specificity for intubation, and could be a warning for exacerbations in critically ill COVID-19 patients [20]. For intubated COVID-19 patients, instead of auscultation, LUS examination can confirm endotracheal intubation by examining lung sliding of both side.

In the early stage of mechanical ventilation in COVID-19, liberal tidal volume and relatively low PEEP levels were recommended [7]. With the disease progress, the protective ventilation strategy with higher PEEP levels may be beneficial. However, inappropriate high PEEP levels will decrease pulmonary compliance and impact right heart function. Performing POCUS to monitor the size of both ventricles and contractility while monitoring lung function, helps to tailor the ventilatory strategy, reaching a balance between lung opening and adequate right heart function [41, 42].

If hypoxaemia progresses to a P/F ratio lower than 150 mmHg, PEEP, recruitment maneuvers (RM) and prone position should be considered [42–44]. Lung RM plus PEEP increases airway pressure to open collapsed alveoli and keep them open throughout the ventilatory cycle. When PEEP is increased stepwisely, lung recruitment regional analysis of independent and nondependent regions may assist the early recognition of responders. Prone position ventilation, that, has the potential to attenuate ventilator-related lung injury and unload the right ventricle while correcting hypoxemia and hypercapnia, should be applied early [45, 46]. At least 12–16 h per day of prone position is strongly recommended for patients with severe ARDS [47, 48]. During these prolonged sessions without transthoracic windows, transesophageal echocardiography is a helpful and necessary tool when these patients undergo persistent respiratory compromise or hemodynamic deterioration with no obvious explanations. Lung ventilation can also be monitored in the lateral and posterior regions of the thorax.

### Extracorporeal membrane oxygenation

Extracorporeal membrane oxygenation (ECMO) is initiated in cases of refractory hypoxemia [49]. V-V ECMO is the primary mode used in COVID-19 patients [44]. However, some patients with COVID-19 have respiratory failure integrated with cardiovascular system damage and circulatory failure, and V-A ECMO is used in this situation. Echocardiographic examination is necessary before catheterization, because V-A ECMO instead of V-V ECMO may be a better choice, when severe left heart dysfunction presents [50]. During catheterization, vascular ultrasound enhances the safety and efficacy with real-time guidance. Improper placement of the cathetermay increase recirculation and reduce the oxygenation efficiency of ECMO. Therefore, ultrasound should be performed after peripheral cannulation to confirm the correct position of the catheter [51]. Additionally, POCUS also plays an important role in the daily management of ECMO patients. POCUS is a valid cardiac output monitoring complement of thermodilution and pulse contour analysis [52, 53].

### Weaning and extubation

For patients gradually recovering respiratory function, earlier liberation from invasive mechanical ventilation reduces the incidence of ventilator-associated complications. However, the risks of premature extubation and subsequent reintubation cannot be avoided completely, which would cause additional harm to patients and increase the risk of viral exposure in healthcare workers. One argument surrounding the challenges of primary extubation shows that COVID-19 patients had a higher chance of re-intubation than non-COVID-19 patients [54]. Evaluation of weaning indications in patients undergoing invasive mechanical ventilation remains a great challenge. POCUS functions as a comprehensive method to predict failed extubation through cardiac, lung, and diaphragmatic dysfunction parameters [55]. There is a positive correlation between echocardiographic measurements of diastolic dysfunction, particularly E/E′*,* and weaning failure [56, 57]. A study suggested that patients are more likely to failed weaning when the left ventricular ejection fraction is lower than 35% and E/E′ is greater than 7.8 [56]. LUSS may be a simple tool to accurately predict post-extubation distress based on a spontaneous breathing trial (SBT) [58, 59]. An end-SBT LUSS < 13 predicted extubation success, and an end-SBT LUSS > 17 predicted postextubation distress [58]. Active diaphragm contraction may increase aeration of posterior and dependent lung regions, promoting extubation success. Diaphragmatic function indices, including diaphragmatic excursion and thickening fraction, can also be rapidly obtained using POCUS [55].

Percutaneous tracheostomy is a common procedure of modern critical care. It benefits patients who require prolonged ventilation, allowing for a more controlled wean. Considering that patients with COVID-19 typically have longer periods of ventilation than patients with other viral pneumonias [60], the use of tracheostomy to aid weaning from ventilatory support should be considered [61], which may ease the burden upon critical care resources [62]. Angel et al. showed that early tracheostomy in COVID-19 patients can be performed with improved patient prognosis [63]. Reported rates of tracheostomies during the coronavirus pandemic were significantly higher than those of pre-pandemic [61, 64, 65]. Ultrasound scan facilitates clinicians to identify thyroid glands, and vessels anterior to trachea, to delineate the airway, to evaluate the thickness of the skin over the neck and even to visualize the needle and guides wire passage, thereby reducing the occurrence of fatal bleeding, pneumothorax, tracheoesophageal fistula and other complications [66]. In addition, ultrasound enables bedside assessment some tracheostomy-associated complications. A study reported that ultrasonography was the first choice for diagnosis of the post-tracheostomy pneumothorax [67].

### Management of severe complications

The most common echocardiographic abnormality among patients with COVID-19 infection was RV dilatation and dysfunction [28], suggesting the possibility of acute cor pulmonale (ACP). ACP is a severe complication defined by RV acute dilatation with paradoxical septal motion during the end-systole in the context of acute lung disease, including ARDS and associated pulmonary vascular dysfunction [68]. The prevalence of ACP ranges from 20 to 25% in the protective mechanical ventilation era, and severe ACP is associated with a poor outcome [40]. In a large prospective observational study, Mekontso Desapp and colleagues reported that the mortality did not differ between patients with or without ACP, but that in patients with severe ACP, it was an independent predictor of mortality [69]. This could be explained by the fact that patients with mildly dilated RV may have preserved RV systolic function [69]. Real time echocardiography is useful to promptly detect mild ACP and guide interventions, including ventilatory strategy adjustment, prone positioning initiation and nitric oxide inhalation to avoid further deterioration of right ventricular function [41, 70].

It is worth noting that acute pulmonary embolism (APE) is also a potential cause of ACP in COVID-19 patients [32, 71]. In the early stage of outbreak, elevated levels of D-dimer in COVID-19 patients attracted attentions [25]. In a study involving 58 COVID-19 patients, 14.8% had DVT despite accepting low-molecular-weight heparin prophylactic treatment [72]. When dilated right ventricule is detected, APE should be considered, and venous ultrasound should be performed, especially in ARDS patients who have sudden hemodynamic instability and severe hypoxemia. But we should be vigilant that negative DVT does not necessarily rule out APE. Patients with APE often have pulmonary hypertension. The pulmonary artery systolic pressure is most commonly obtained by a modified Bernoulli equation to convert peak tricuspid regurgitation velocity into pressure and to add the right atrial pressure. Additionally, in the absence of reliable tricuspid regurgitant signals, the pulmonary ejection signal acceleration time (PACT) also can be used to assess pulmonary artery pressure. For example, a PACT of 70–90 ms indicates a pulmonary artery systolic pressure > 70 mmHg [73].

### Hemodynamics and fluid administration

Significant hemodynamic derangement can accompany COVID-19-related ARDS. In a large cohort of COVID-19 patients, the incidence of circulatory shock was 30% [74]. Identifying the etiology and mechanism of shock is important to select the best therapy. These patients are initially resuscitated with fluid, and POCUS should be performed if hemodynamic instability persists.

Fluid resuscitation is essential in patients with shock. For example, severe hemorrhagic shock can lead to outflow tract obstruction if the patient is under-resuscitated, especially for patients with the LV exhibits significant asymmetric septal, or severe concentric, hypertrophy. However, some studies reported that positive fluid balance is an independent risk factor for ARDS development [75]. RV failure can be precipitated if excessive fluid resuscitation is undertaken which, in turn, may cause shifting of the interventricular septum toward the left impeding LV diastolic filling [76]. This would lead to pulmonary edema and hypoxemia which will further worsen RV failure, thus forming a vicious circle. The optimal fluid management for ARDS patients is key to restore adequate organ perfusion while avoiding diffuse tissue edema and positive fluid balance [77]. LUS is sensitive to pulmonary edema with the pattern of “comet tails”, which functions as a “safeguard” against excessive fluid by dynamic evaluation. It can be used along with simple cardiac and vena cava analysis for fluid resuscitation administration [78].

Inferior vena cava (IVC) ultrasound examination should be performed as the first step to identify the etiology of shock [79]. However, there are limitations to use the IVC alone to estimate mean systemic venous pressure. In order to identify the etiology of shock, it is also necessary to examine the heart. Doppler examination of the hepatic venous flow can also be added [80]. In patients with reduced/increased mean systemic venous pressure, further ultrasound examination of the thoracic and abdominal should be considered to detect free fluid, empyema, pneumothorax, or cardiac tamponade. Subsequent treatment can be tailored according to the etiology of shock.

## Conclusion

SARS-CoV-2 can cause bilateral pneumonia. Among those with pneumonia, 20–42% have developed ARDS [81], which seriously affects COVID-19 prognosis. POCUS is a rapid, feasible, and safe bedside tool for respiratory and hemodynamic evaluation of patients with COVID-19. This review provides information about the use of POCUS in COVID-19 patients with ARDS. It might help intensivists optimize and make individualized management for these patients.

## Data Availability

Not applicable.

## Abbreviations

COVID-19: Coronavirus disease 2019
SARS-CoV-2: Severe acute respiratory syndrome coronavirus 2
WHO: World Health Organization
ARDS: Acute respiratory distress syndrome
POCUS: Point-of-care ultrasound
ICU: Intensive care unit
CT: Computed tomography
PEEP: Positive end expiratory pressure
ECMO: Extracorporeal membrane oxygenation
LUS: Lung ultrasound
RM: Recruitment maneuvers
LUSS: Lung ultrasound score
SBT: Spontaneous breathing trial
ACP: Acute cor pulmonale
RVEDA: The right ventricular end-diastolic area
LVEDA: The left ventricular end-diastolic area
APE: Acute pulmonary embolism
DVT: Deep vein thrombosis
RV: Right ventricular
LV: Left ventricular
AKI: Acute kidney injury
IVC: Inferior vena cava
